# Integrated gut microbiome and metabolomics analysis reveals microbial-metabolic cross-talk in allergic rhinitis

**DOI:** 10.3389/fmicb.2025.1652915

**Published:** 2025-11-12

**Authors:** Guangchen Sun, Shouyan Zhao, Hehua Huang, Wenchao Guan, Xinzhuo Wang, Hong Zhang, Min Zhang, Denghan Hou, Chong Xu, Ruonan Chai

**Affiliations:** 1 Department of Respiratory Medicine, General Hospital of Northern Theater Command, Shenyang, Liaoning, China; 2 College of Medicine and Biological Information Engineering, Northeastern University, Shenyang, Liaoning, China; 3 Department of Respiratory Medicine, The 962nd Hospital of the PLA Joint Logistic Support Force, Harbin, Heilongjiang, China

**Keywords:** allergic rhinitis, gut microbiome, 16S rRNA gene sequencing, untargeted metabolomics, gut-nose axis

## Abstract

**Background:**

Emerging evidence indicates a link between gut dysbiosis and allergic rhinitis (AR) pathogenesis. Nevertheless, the mechanistic role of gut microbiota in AR progression requires further characterization. To address this, we employed an integrated multi-omics strategy to delineate gut microbial composition and metabolic signatures in AR patients.

**Methods:**

Fecal specimens from 23 AR patients and 15 matched healthy controls (total *n* = 38) were subjected to 16S rRNA gene sequencing to assess bacterial community structure, alongside untargeted metabolomic profiling of microbial metabolites. Spearman’s rank correlation analysis was applied to evaluate microbiota-metabolite interactions.

**Results:**

Allergic rhinitis patients exhibited altered gut microbial community structure (beta diversity, *P* < 0.05) with depletion of SCFA-producing genera such as *Faecalibacterium* and enrichment of pro-inflammatory taxa like *Fusobacterium*. Metabolomic profiling identified significant disturbances in pathways including pantothenate and CoA biosynthesis, glycolysis, and pyruvate metabolism. Key discriminatory metabolites included maltol and 4-coumaric acid. Integrative analysis revealed significant correlations between specific bacteria and metabolites, such as *Faecalibacterium* with D-phenyllactic acid (ρ = 0.515, *q* = 0.046).

**Conclusion:**

Our findings demonstrate that AR is associated with gut dysbiosis and metabolic dysfunction, highlighting the role of microbial-derived metabolites in immune regulation via the gut-nose axis. These insights support the potential for microbiota-targeted therapeutic strategies in AR management.

## Introduction

Allergic rhinitis (AR) is the most common allergic disease worldwide and one of the most common chronic diseases in general, imposing substantial socioeconomic burdens due to its chronicity and association with comorbidities such as asthma and sinusitis (Bousquet et al., 2020; Patel et al., 2024; Zhang et al., 2021). AR is a non-infectious chronic inflammatory disease of the nasal mucosa, clinically characterized by paroxysmal sneezing, watery rhinorrhea, nasal pruritus, nasal congestion, and commonly accompanied by ocular pruritus and other allergic mucosal manifestations, with a concomitant asthma prevalence rate of approximately 35.7%–48.5% among AR patients (Sousa-Pinto et al., 2025). The pathogenesis of AR classically involves Th2-driven IgE sensitization, mast cell degranulation (histamine, leukotrienes), and eosinophil-mediated inflammation, whereas emerging evidence underscores the pivotal role of epithelium-derived DAMPs (damage-associated molecular patterns, such as TSLP, IL-25, and IL-33) in activating type 2 innate lymphoid cells (ILC2s), alongside microbial dysbiosis, neuroimmune interactions, and disease endotype stratification (He et al., 2024; Nian et al., 2020; Yang M. et al., 2023; Zhang et al., 2022; Zoabi et al., 2022). Despite advances in understanding its pathophysiology (Drazdauskaitë et al., 2020; Liu et al., 2022; Wang et al., 2023a; Zheng and Yu, 2022), the exact mechanisms driving the development of AR have not been fully elucidated (Gerth van Wijk and Smits, 2021; Wise et al., 2023).

The gut microbiota plays a pivotal role in modulating systemic immune homeostasis and inflammatory cascades; emerging evidence highlights that perturbations in microbial richness, taxonomic diversity, community structure, and microbial-derived metabolites are mechanistically linked to the development and progression of multiple allergic pathologies (Jin et al., 2023; Ke et al., 2025; Sasaki et al., 2024; Wang et al., 2023c). The gut microbiota exerts multifaceted influences on the pathogenesis and progression of AR through mechanisms encompassing immune modulation, maintenance of epithelial barrier integrity, and regulation of inflammatory responses (Aguilera et al., 2020; Chiu et al., 2019; Ding et al., 2025; Liu et al., 2024). This systemic influence is encapsulated within the broader framework of interconnected mucosal immunity, such as the skin-gut-lung axis, which underscores the role of microbial dysbiosis at one site in influencing allergic inflammation at remote organs, including the nasal mucosa (Yang et al., 2025). Key microbial-derived metabolites mediate these effects, including short-chain fatty acids (SCFAs) and polyamines that demonstrate potent immunomodulatory properties, tryptophan catabolites (indole derivatives) that activate the aryl hydrocarbon receptor (AhR) signaling pathway, and secondary bile acids (BAs) exhibiting anti-inflammatory capacities (Chen et al., 2022; Park et al., 2015, 2018; Roduit et al., 2019; Wang et al., 2024; Zhou C. J. et al., 2021). Specifically in the context of nasal inflammation, a recent systematic review consolidates evidence for gut microbiota alterations in AR patients, reinforcing the concept of a gut-nose axis, albeit with heterogeneity in specific taxa identified across studies (Hu et al., 2024). This axis is further supported by findings in chronic rhinosinusitis (CRS), where patients exhibit indicator gut microbiota alterations (e.g., reduced *Faecalibacterium* and *Bifidobacterium*), suggesting a shared gut-sinus relationship across different forms of sinonasal inflammation (Michalik et al., 2023). The mechanistic underpinnings of this axis may involve microbial translocation and immune crosstalk, as hypothesized in evolving models where pathogens like *Staphylococcus aureus* could traverse from the sinus to the gut and vice versa, potentially exacerbating inflammation (Jad, 2024). Notably, the therapeutic potential of gut microbiota-targeted interventions in AR has garnered preliminary validation through a growing body of evidence from both preclinical animal models and human clinical trials (Dong et al., 2024; Galvan Calle et al., 2022; Hou et al., 2024; Lungaro et al., 2024; Zhou et al., 2024). However, the causal links between microbial dysbiosis and disease progression—particularly how microbiota-derived metabolites mediate immune dysregulation via the gut-nose axis—await systematic exploration.

To address these knowledge gaps, we performed a multi-omics analysis of fecal samples from AR patients and healthy controls, combining 16S rRNA gene sequencing with untargeted metabolomics. Our study aims to: (1) identify AR-specific alterations in gut microbial composition and metabolic pathways; (2) characterize correlations between dysbiotic taxa and immunomodulatory metabolites. By integrating microbial taxonomy with functional metabolomics, this work provides a comprehensive perspective on the gut-nose axis in AR and identifies potential targets for microbiota-based therapeutics.

## Materials and methods

### Study design

Participants in this study were recruited from an ongoing study supported by the Natural Science Foundation of Liaoning Province, China (Grant No. 2022JH2/101500014), titled “Pathogenesis of intestinal flora dysbiosis in allergic rhinitis and the application of washed microbiota transplantation in AR treatment” (2022–2025). A total of 23 patients met the criteria of sample collection, and agreed to join in this study (Group A). In addition, 15 age- and sex-matched healthy controls were recruited from the community (Group B). All participants signed the written informed consent. The following demographic and clinical data were collected via in-person interviews: age, sex, weight, height, body mass index (BMI), educational attainment, smoking history, alcohol consumption history, marital status, family medical history, disease duration, medication and dietary supplement usage, Total Nasal Symptom Score (TNSS), and Rhinoconjunctivitis Quality of Life Questionnaire (RQLQ) outcomes. This study protocol was conducted in accordance with the Declaration of Helsinki and approved by the Ethical Committee of General Hospital of Northern Theater Command of PLA (Approval number: Y(2025)092).

### Study population

The diagnosis of AR was based on ARIA guidelines (2016 revision), Chinese Society of Allergy Guidelines for Diagnosis and Treatment of Allergic Rhinitis (2018) and Chinese guideline for diagnosis and treatment of allergic rhinitis (2022 revision) (Brożek et al., 2017; Cheng et al., 2018; Subspecialty Group of Rhinology, Editorial Board of Chinese Journal of Otorhinolaryngology Head and Neck Surgery, and Subspecialty Group of Rhinology, Society of Otorhinolaryngology Head and Neck Surgery, Chinese Medical Association, 2022). The inclusion criteria of Group A were as follows: (1) paroxysmal sneezing, clear water-like runny nose, itch, sneezing, and other symptoms appear 2 or more, and the daily symptoms persist or accumulate more than 1 h; (2) the presence of allergen-specific IgE antibody (sIgE) test ≥0.35 IU/mL and/or positive skin prick tests (SPT); (3) a history of a reaction in the past year; (4) age 18–65 years; (5) voluntary participation in this study. In addition, 15 age- and sex-matched healthy controls were recruited from the community. The inclusion criteria of Group B were as follows: (1) there is no history of allergies or family allergies;(2) there are no allergy-related symptoms;(3) voluntary participation in this study. Exclusion criteria for both groups included: (1) receipt of systemic or topical antibiotics, immunomodulatory agents (including glucocorticoids), antihistamines, probiotics, prebiotics, or synbiotics within 3 months prior to enrollment; (2) use of laxatives or antidiarrheal medications, or experience of constipation, diarrhea, or respiratory tract infection within the preceding 4 weeks; (3) comorbid respiratory conditions including chronic obstructive pulmonary disease, asthma, bronchiectasis, tuberculosis, pneumonia, pulmonary heart disease, or pulmonary malignancies; (4) history of hypertension, coronary heart disease, hyperthyroidism, hypothyroidism, hepatic or renal dysfunction, or hematologic disorders; (5) history of psychiatric or neurological conditions; (6) presence of clinically significant abnormalities upon pre-trial assessment deemed likely to confound study outcomes, as determined by the investigators (Liu et al., 2020; Zhou M. S. et al., 2021; Zhu et al., 2020). All study participants shared comparable ethnic/geographic and dietary backgrounds.

### TNSS and RQLQ

To comprehensively evaluate the symptom severity and quality of life impact of AR, we utilized two validated clinical tools: TNSS and RQLQ. The TNSS assesses the severity of four key nasal symptoms: nasal obstruction, rhinorrhea, sneezing, and nasal itching. Each symptom was scored by patients on a 4-point Likert scale: 0 = no symptoms; 1 = mild symptoms (present but not bothersome); 2 = moderate symptoms (noticeable and occasionally bothersome); 3 = Severe symptoms (frequent and significantly bothersome). The total TNSS ranged from 0 to 12, with higher scores indicating greater symptom severity. The RQLQ is a disease-specific instrument designed to measure the impact of AR on patients’ quality of life. The questionnaire comprises 28 items across seven domains: sleep disturbances, nasal symptoms, ocular symptoms, practical problems, emotional function, activity limitations, and general well-being. Each item was rated on a 7-point scale (0–6), where: 0 = No impairment; 6 = Severe impairment. The overall RQLQ score was calculated as the mean of all item scores, yielding a total range of 0 to 6, with higher scores reflecting worse quality of life (QoL). Both TNSS and RQLQ have demonstrated high reliability and validity in prior studies of AR (Bousquet et al., 2025; Sánchez and Castro, 2019), making them suitable for capturing symptom burden and its functional consequences in our cohort.

### Fecal sample collection

Fecal samples were collected from all participants by retaining the mid-to-late portion of bowel movements. Using sterilized spoons, the inner layer of fecal material was carefully sampled, and all specimens were transferred into sterile plastic tubes under aseptic conditions, with precautions taken to avoid contamination from urine or contact with toilet surfaces. Additionally, all female participants provided stool samples exclusively during their non-menstrual phase. Within 2 h post-collection, samples were transported to the laboratory in ice-packed coolers to maintain a cold chain, followed by immediate storage at −80°C until subsequent analyzes.

### Gut microbiome detection and analysis

Fecal samples from AR patients and healthy controls were collected using sterile protocols, stored at −80°C, and processed for 16S rRNA gene sequencing. Genomic DNA was extracted (QIAamp DNA Stool Mini Kit), and the V3-V4 region was amplified with primers 357F/806R using a two-step PCR protocol (Phusion polymerase). Libraries were sequenced on Illumina NovaSeq (250-bp paired-end). Bioinformatic analysis included quality filtering [Trimmomatic (Bolger et al., 2014)], amplicon sequence variant (ASV) clustering using the DADA2 (Callahan et al., 2016) pipeline, chimeric removal (integrated within DADA2), and taxonomic annotation (SILVA 138). Alpha diversity indices (Observed species, Chao1, ACE, Shannon, Simpson, and Phylogenetic Diversity whole tree) and beta diversity metrics (Bray-Curtis, Jaccard, unweighted and weighted UniFrac) and LEfSe (Segata et al., 2011) (LDA > 2, *P* < 0.05) were calculated using QIIME 2 (Bolyen et al., 2019).

### Metabolomics detection and analysis

Fecal metabolites were profiled via UHPLC-QTOF-MS (Agilent 6545). Samples were homogenized in 80% methanol, centrifuged, and filtered. Raw data were processed with XCMS for peak alignment, normalization, and QC-based filtering (RSD < 30%). Multivariate analysis included PCA and OPLS-DA (SIMCA-P, validated by permutation tests). The OPLS-DA model was used strictly as an exploratory tool for variable selection and not for predictive purposes. Differential metabolites were identified (VIP > 1.0, FC ≥ 1.5/ ≤ 0.667, *P* < 0.05) and mapped to KEGG pathways [MetaboAnalyst 5.0 (Pang et al., 2021)] using hypergeometric tests. For the receiver operating characteristic (ROC) analysis of candidate metabolites, internal validation was performed using a 10-fold cross-validation scheme.

### Statistical analysis

Statistical analysis was performed using R software (version 4.0.2). For baseline characteristics of study participants, continuous variables were first assessed for normality using the Kolmogorov-Smirnov test. Normally distributed variables were compared between groups using Independent *t*-test, while non-normally distributed variables were analyzed with Mann-Whitney U test to assess median differences. Categorical variables were evaluated using Fisher’s exact test considering the limited sample size. Difference of community structure of gut microbiome among groups was analyzed using the method of permutational multivariate ANOVA (PERMANOVA). Metabolomic profiles were processed with log-transformation prior to statistical analysis. Differentially expressed metabolites were identified using MS/MS spectral data and verified through non-parametric statistical testing (Mann-Whitney U). Spearman’s rank correlation analysis was conducted to explore associations between gut microbiota and metabolites, with false discovery rate (FDR) correction applied using Benjamini-Hochberg procedure to adjust for multiple comparisons. All reported *p*-values were two-tailed and *P* < 0.05 was considered significant.

## Results

### Characteristics of study populations

The study cohort comprised 38 participants, including 23 AR patient (Group A) and 15 healthy controls (Group B). Demographic and anthropometric characteristics were comparable between groups (Table 1). The AR group (median age: 37 years, IQR: 31–42) and controls (median age: 35 years, IQR: 26–43) showed no significant differences in age (*P* = 0.58, Mann-Whitney U test) or gender distribution (47.8% vs. 40.0% males, *P* = 0.65, χ^2^ test). BMI was marginally higher in AR patients (mean ± SD: 23.8 ± 3.1 kg/m^2^) than in controls (21.8 ± 3.5 kg/m^2^), though this difference was not statistically significant (*P* = 0.12, independent *t*-test). Ethnic homogeneity was observed, with Han Chinese constituting 82.6% of AR patients and 93.3% of controls (*P* = 0.35, Fisher’s exact test).

**TABLE 1:** Baseline data and clinical characteristics of the subjects.

Variable	Group A (n = 23)	Group B (n = 15)	*P*-value
Age, years	37.0 ± 10.2	35.3 ± 12.5	0.58
Gender, n (%)			0.65
Male	11 (47.8)	6 (40.0)	
Female	12 (52.2)	9 (60.0)	
BMI, kg/m^2^	23.8 ± 3.1	21.8 ± 3.5	0.12
Ethnicity, n (%)			0.35
Han Chinese	19 (82.6)	14 (93.3)	
Other	4 (17.4)	1 (6.7)	
Smoking, years	0 [0–3]	0 [0–5]	0.62
Alcohol use, years	0 [0–10]	0 [0–7]	1.00
Disease duration, months	12 [6–19]	N/A	–
TNSS	5 [4–8]	N/A	–
RQLQ	42 [32–61]	N/A	–

Normally distributed variables are expressed as mean ± SD, while non-normally distributed variables are reported as median [interquartile range, IQR], based on Kolmogorov-Smirnov test results (α = 0.05). TNSS, Total Nasal Symptom Score; RQLQ, Rhinoconjunctivitis Quality of Life Questionnaire.

Lifestyle factors and clinical parameters further defined the cohorts. Smoking and alcohol consumption were infrequent in both groups: 8.7% of AR patients (median smoking duration: 3 years, IQR: 3–12) and 13.3% of controls (median: 5 years, IQR: 5–5) reported smoking (*P* = 0.62), while alcohol use was reported by 13.0% of AR patients (median: 10 years, IQR: 7–15) and 13.3% of controls (median: 7 years, IQR: 7–7, *P* = 1.00). Symptom severity and quality of life impact were quantified by the Total Nasal Symptom Score (TNSS: 5, IQR: 4–8) and Rhinoconjunctivitis Quality of Life Questionnaire (RQLQ: 42, IQR: 32–61), respectively, reflecting clinically relevant allergic burden.

### Characteristics of gut microbiome

Quality control of sequencing data revealed a unimodal distribution of effective sequence lengths, with a median of 420 bp (IQR: 414.5–419.5 bp), indicating high consistency across samples (Supplementary Figure 1). Rarefaction curves approached asymptote at a sequencing depth of 40,000 reads per sample (Supplementary Figure 2), indicating that the majority of microbial diversity within each sample was effectively captured. This confirms that the sequencing effort was sufficient for robust downstream analyses of alpha and beta diversity. Although the species accumulation curve did not reach a complete plateau, the rate of new species discovery markedly decreased after approximately 20 samples (Supplementary Figure 3), indicating that our cohort size was sufficient to capture the majority of the microbial diversity.

**FIGURE 1:**
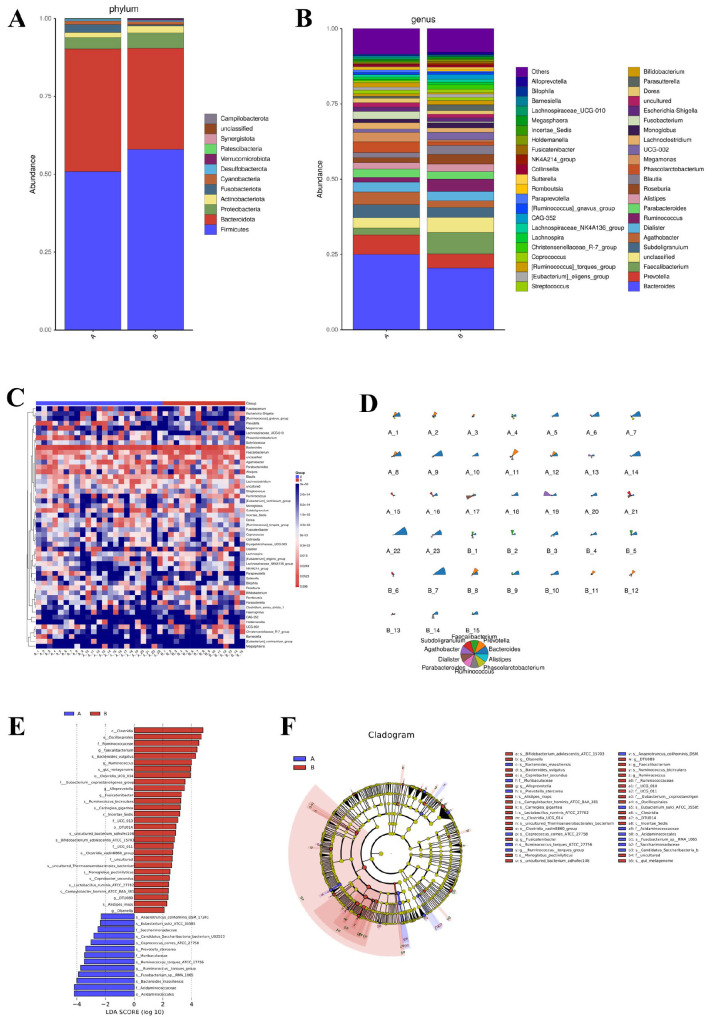
Gut microbial composition and taxonomic differences between AR patients and healthy controls. **(A)** Bar plot showing the relative abundance of bacterial phyla. **(B)** Bar plot showing the relative abundance of bacterial genera. **(C)** Heatmap depicting hierarchical clustering of samples based on Z-score normalized genus-level abundances. **(D)** Star plot illustrating the distribution of the top 10 most abundant genera in each group. **(E)** Histogram of Linear Discriminant Analysis (LDA) scores for taxa with significant differences between groups (LDA score > 2.0, *P* < 0.05 by Kruskal-Wallis test). **(F)** Cladogram generated by LEfSe analysis, showing the phylogenetic distribution of discriminative taxa from phylum to genus level. The LEfSe analysis uses a non-parametric factorial Kruskal–Wallis sum-rank test followed by LDA.

The gut microbiota composition of AR patients (Group A, *n* = 23) and healthy controls (Group B, *n* = 15) exhibited significant differences at both phylum and genus levels. At the phylum level (Figure 1A), Firmicutes and Bacteroidetes were dominant in both groups. However, AR patients exhibited a marked increase in the relative abundance of the pro-inflammatory phylum Fusobacteriota, alongside a significant reduction in the SCFA-producing phylum Verrucomicrobiota.

Analysis at the genus level (Figure 1B) revealed that *Faecalibacterium*, a keystone SCFA-producing genus enriched in healthy individuals, was significantly depleted in AR patients. Other beneficial SCFA producers, including *Ruminococcus* and *Roseburia*, also showed a tendency toward reduced abundance in the AR group. Conversely, genera with potential pro-inflammatory associations demonstrated an opposite trend: *Fusobacterium*, which was nearly absent in HC, was present at high abundance in AR patients. Genus-level heatmap analysis (Figure 1C) and a star plot of the top 10 most abundant genera (Figure 1D) further confirmed distinct clustering patterns between AR patients and HC, reflecting clear differences in microbial community structure.

Linear discriminant analysis (Figure 1E) identified *Clostridia*, *Ruminococcaceae*, and *Faecalibacterium* as key discriminators for HC (LDA score > 3.0), while *Synergistetes* and *Bacillales* were enriched in AR. Phylogenetic cladogram analysis (Figure 1F) further substantiated these systematic differences from an evolutionary perspective. HC exhibited enrichment of SCFA-producing families such as *Ruminococcaceae* and *Lachnospiraceae*, whereas AR patients showed overrepresentation of potentially pathogenic bacteria, including *Collinsella*. Collectively, these findings indicate that AR is associated with gut microbial dysbiosis, characterized by a decline in SCFA-producing bacteria and an expansion of pro-inflammatory taxa, which may contribute to disease pathogenesis.

Alpha diversity analysis revealed no statistically significant differences in gut microbial richness and diversity between AR patients and HC (Supplementary Figure 4). Indices reflecting microbial richness—including Observed species (*P* = 0.174), Chao1 (*P* = 0.174), and ACE (*P* = 0.181)—and indices representing microbial diversity, such as Shannon (*P* = 0.191) and Simpson (*P* = 0.145), all showed *P*-values greater than 0.05. Notably, the phylogenetic diversity index phylogenetic diversity whole tree (PD whole tree) approached statistical significance (*P* = 0.051), suggesting a potential tendency toward reduced phylogenetic complexity of the gut microbiota in AR patients compared with healthy individuals, though this trend did not reach the conventional significance threshold (*P* < 0.05). Collectively, these findings indicate that the overall taxonomic breadth of the gut microbial community does not differ substantially between AR patients and healthy controls, while the evolutionary composition of the microbiota may have a subtle difference that requires further verification with a larger sample size.

**FIGURE 2:**
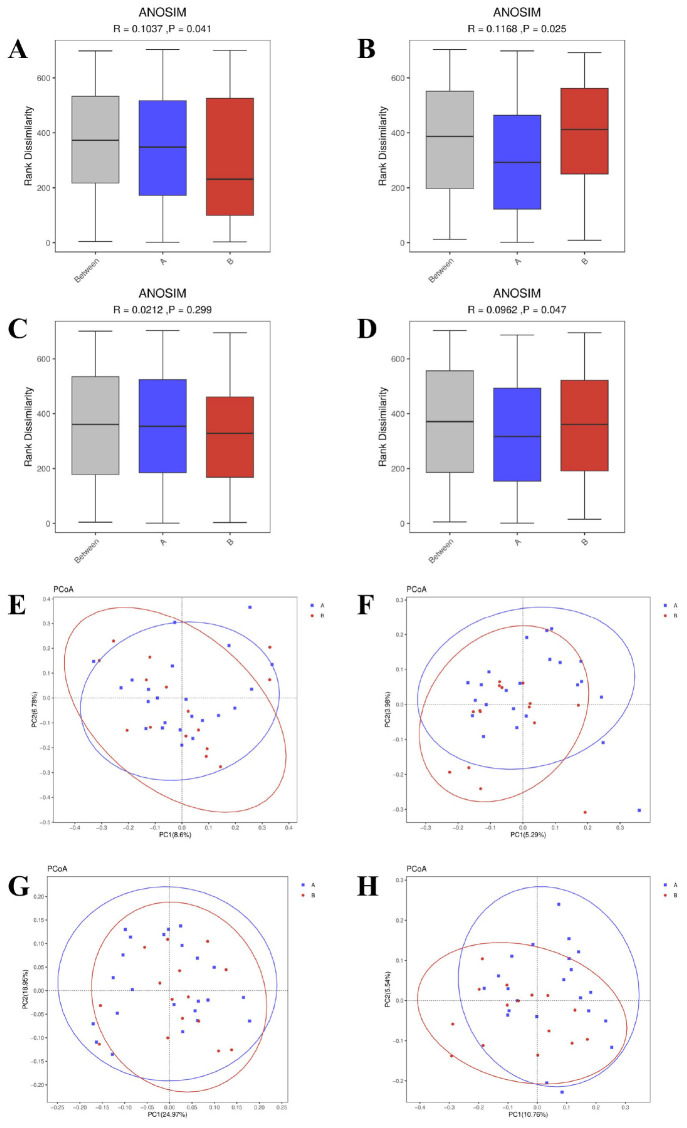
Beta diversity analysis of gut microbiota between AR patients (Group A) and healthy controls (Group B). **(A–D)** Boxplots of between-group dissimilarities based on panel **(A)** Bray-Curtis, **(B)** Jaccard, **(C)** weighted UniFrac, and **(D)** unweighted UniFrac distance metrics. The *P*-values and effect sizes (R^2^) were calculated using Permutational Multivariate Analysis of Variance (PERMANOVA) with 999 permutations. **(E–H)** Principal Coordinate Analysis (PCoA) plots visualizing group separation based on the same distance metrics: **(E)** Bray-Curtis, **(F)** Jaccard, **(G)** weighted UniFrac, and **(H)** unweighted UniFrac. The percentage of variance explained by each principal coordinate is indicated on the axes.

Beta diversity analysis highlighted differences in microbial community structures between the AR patients and HC. Using four common distance metrics (Bray-Curtis, Jaccard, weighted UniFrac, and unweighted UniFrac) and PERMANOVA analysis (with effect size represented by R^2^), significant differences were observed in Bray-Curtis distance (*P* = 0.041, R^2^ = 0.043; Figure 2A), Jaccard distance (*P* = 0.025, R^2^ = 0.031; Figure 2B), and unweighted UniFrac distance (*P* = 0.047, R^2^ = 0.039; Figure 2D), while weighted UniFrac distance showed no significant difference (*P* = 0.299, R^2^ = 0.040; Figure 2C). Principal Coordinate Analysis (PCOA) visualization consistently showed that samples based on Bray-Curtis (Figure 2E), Jaccard (Figure 2F), and unweighted UniFrac (Figure 2H) distances exhibited a tendency of separation between the AR patients and HC, whereas the PCOA plot based on weighted UniFrac (Figure 2G) distance showed no obvious group separation. Principal Coordinate 1 (PC1) accounted for 8.6% (Bray-Curtis), 5.29% (Jaccard), 24.97% (weighted UniFrac) and 10.76% (unweighted UniFrac) of the total variance, while Principal Coordinate 2 (PC2) explained 6.78% (Bray-Curtis), 3.98% (Jaccard), 18.95% (weighted UniFrac) and 5.54% (unweighted UniFrac) of the total variance, respectively. These results collectively indicate that AR is associated with alterations in gut microbial community structure, and the compositional dissimilarity between AR patients and HC is mainly driven by the presence/absence of rare taxa rather than the relative abundance of dominant taxa.

### Characteristics of gut metabolomics

**FIGURE 3:**
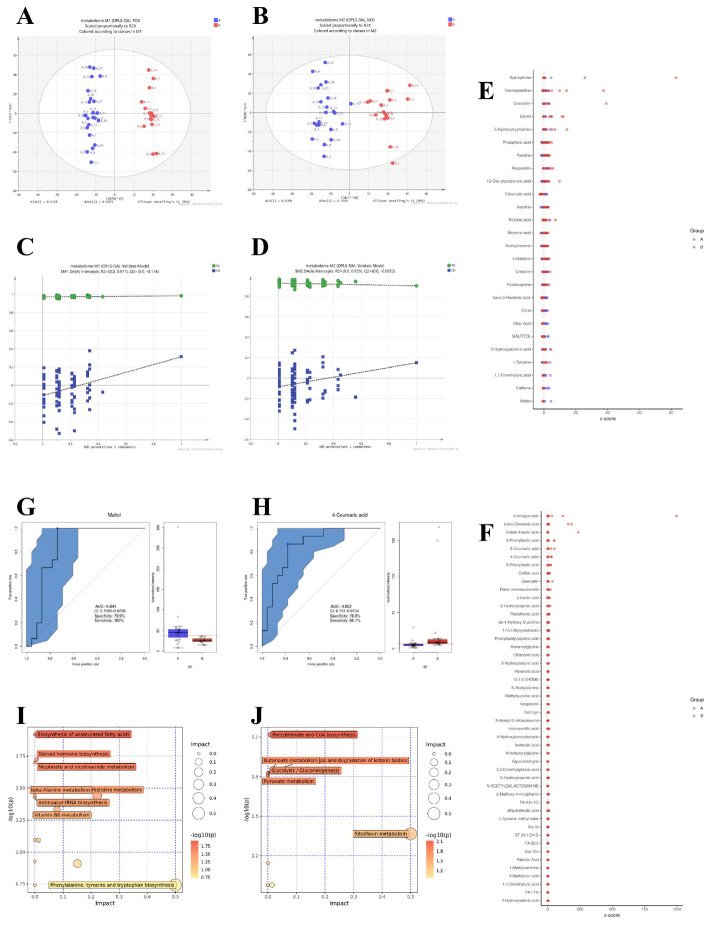
Metabolomic profiling and pathway analysis in AR patients versus healthy controls. **(A,B)** Orthogonal Partial Least Squares-Discriminant Analysis (OPLS-DA) score plots derived from UHPLC-QTOF-MS data in positive (POS, **A**) and negative (NEG, **B**) ionization modes. **(C,D)** Corresponding validation plots from permutation tests (200 permutations). **(E,F)** Heatmaps displaying Z-scores of differentially abundant metabolites in POS **(E)** and NEG **(F)** modes. Differential metabolites were identified based on a Variable Importance in Projection (VIP) > 1.0 from the OPLS-DA model, fold change (FC) ≥ 1.5 or ≤ 0.667, and a *P*-value < 0.05 from the Mann-Whitney U test. **(G,H)** Receiver Operating Characteristic (ROC) curves for the top discriminatory metabolites, Maltol **(G)** and 4-Coumaric acid **(H)**, with Area Under the Curve (AUC), sensitivity, and specificity values indicated. **(I, J)** Summary of pathway enrichment analysis from MetaboAnalyst 5.0 for POS **(I)** and NEG **(J)** modes. The *P*-values for pathway enrichment were calculated using a hypergeometric test and adjusted for multiple comparisons using the Benjamini-Hochberg false discovery rate (FDR) method. Pathways with an FDR-corrected *P*-value (*q*-value) <0.05 were considered significantly perturbed.

The orthogonal partial least squares-discriminant analysis (OPLS-DA) revealed distinct clustering between AR patients and HC in both positive (POS) and negative (NEG) ionization modes (Figures 3A, B). The model demonstrated robust validity, with high explanatory power (R^2^ = 0.97 for POS, R^2^ = 0.94 for NEG) based on permutation testing (Figures 3C, D).

We further identified differentially abundant metabolites between the two groups. In the POS mode (Figure 3E), metabolites such as Epinephrine, Normeperidine, Quercetin, and Maltol exhibited distinct z-scores between AR patients and healthy controls. For instance, Maltol showed a notably higher abundance in AR patients, which was validated by its ROC curve with an AUC of 0.841 [95% CI: 0.706–0.976], 100% sensitivity, and 73.9% specificity (Figure 3G). In the NEG mode (Figure 3F), metabolites including Indolepyruvate, trans-Cinnamic acid, and 4-Coumaric acid were differentially expressed. 4-Coumaric acid had an AUC of 0.852 [95% CI: 0.731–0.973], 86.7% sensitivity, and 78.3% specificity (Figure 3H), indicating its potential as a discriminatory metabolite.

Pathway enrichment analysis highlighted significant perturbations in metabolic networks associated with AR. In the POS mode (Figure 3I), pathways such as biosynthesis of unsaturated fatty acids, steroid hormone biosynthesis, and nicotinate and nicotinamide metabolism were significantly impacted. In the NEG mode (Figure 3J), key pathways included pantothenate and CoA biosynthesis, glycolysis/gluconeogenesis, and pyruvate metabolism, suggesting alterations in energy metabolism and vitamin biosynthesis in AR patients.

### Correlation between gut microbiome and metabolites

To comprehensively assess the interactions between gut microbiota and metabolites in AR patients and HC, we performed Spearman correlation analysis across multiple metabolite classes, including amino acids, lipids, nucleotides, peptides and other metabolites (e.g., carbohydrates, energy-related compounds, vitamins and cofactors). All *p*-values were adjusted for multiple comparisons using the false discovery rate (FDR) method, and only FDR-corrected *p*-values (*q*-values) are reported. Correlations with *q* < 0.05 were considered statistically significant, while those approaching significance (*q* < 0.10) are discussed as suggestive trends.

**FIGURE 4:**
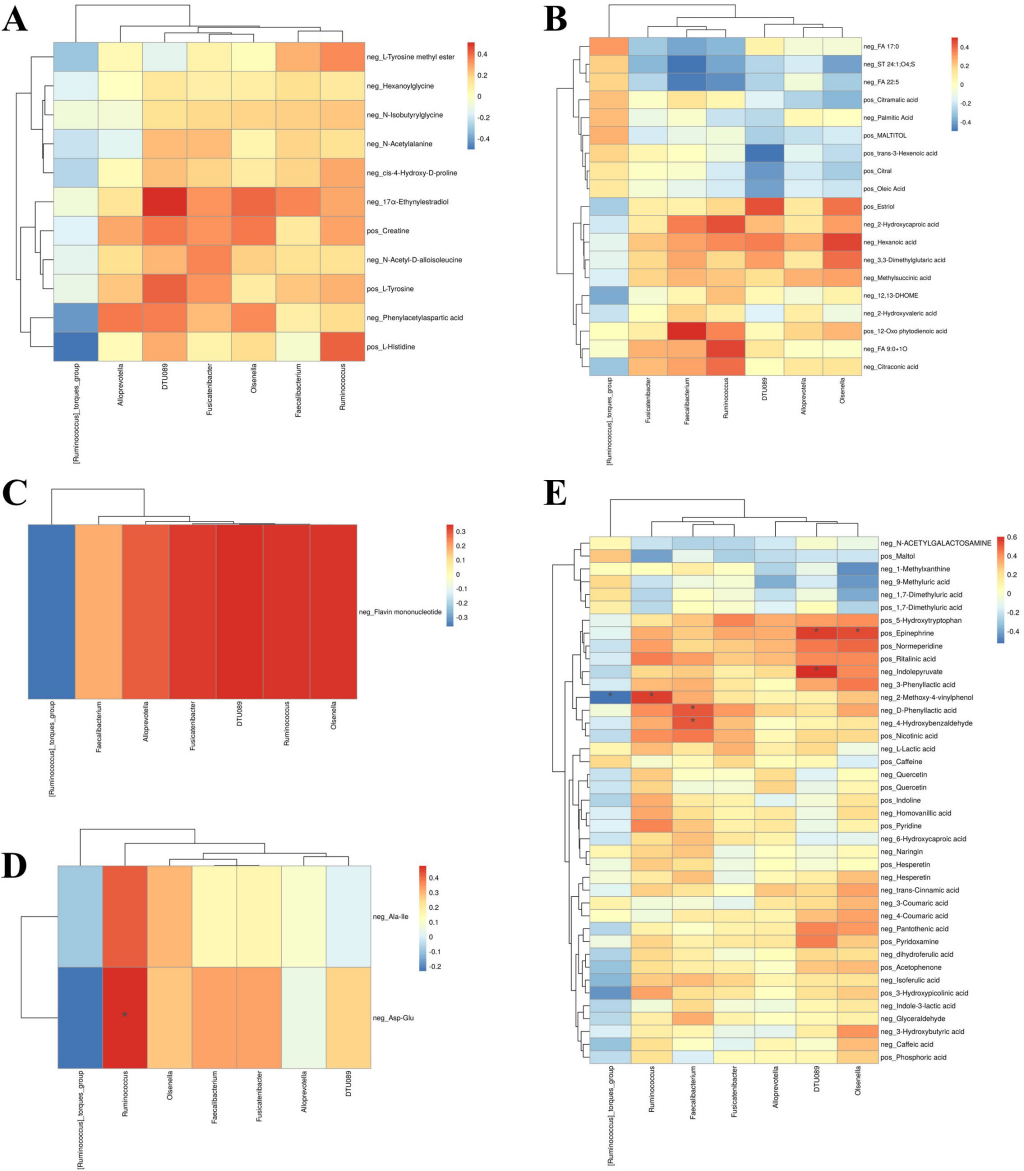
Correlation network between gut microbiota and fecal metabolites. Spearman’s rank correlation analysis between significantly altered microbial genera and metabolites categorized as **(A)** amino acids, **(B)** lipids, **(C)** nucleotides, **(D)** peptides, and **(E)** other metabolites (e.g., carbohydrates, vitamins, phenolic compounds). *P*-values were adjusted for multiple comparisons using the Benjamini-Hochberg false discovery rate (FDR) method. Correlation coefficients and FDR-corrected *q*-values are indicated in the heatmaps. A correlation was deemed statistically significant if the *q*-value was <0.05.

Significant correlations were observed primarily for peptides and other metabolites. Among peptides (Figure 4D), the dipeptide Asp-Glu showed a strong positive correlation with *Ruminococcus* (ρ = 0.481, *q* = 0.031). Among other metabolites (Figure 4E), significant associations included positive correlations of D-phenyllactic acid with *Faecalibacterium* (ρ = 0.515, *q* = 0.046), 2-methoxy-4-vinylphenol with Ruminococcus (ρ = 0.565, *q* = 0.021), 4-hydroxybenzaldehyde with *Faecalibacterium* (ρ = 0.514, *q* = 0.046), and epinephrine with *Olsenella* (ρ = 0.537, *q* = 0.037). A significant negative correlation was observed between 2-methoxy-4-vinylphenol and *Ruminococcus torques* (ρ = −0.526, *q* = 0.040). No significant correlations were found for amino acids, lipids, or nucleotides after FDR correction.

Although amino acids, lipids and nucleotides did not yield statistically significant results, several trends were noted. Amino acid (Figure 4A), 17α-Ethynylestradiol was positively associated with *Olsenella* (ρ = 0.405, *q* = 0.149). Among lipids (Figure 4B), 2-Hydroxycaproic acid showed positive correlations with *Ruminococcus* (ρ = 0.440, *q* = 0.096), and 12-Oxo phytodienoic acid was positively associated with *Faecalibacterium* (ρ = 0.503, *q* = 0.084). Nucleotides (Figure 4C), such as flavin mononucleotide, showed positive trends with multiple genera including *Ruminococcus* (ρ = 0.340, *q* = 0.061).

In summary, this integrative analysis revealed significant microbe-metabolite interactions primarily involving peptides and other metabolites, particularly those related to microbial metabolism of aromatic compounds, neurotransmitters, and phenolic acids. These findings suggest a specific role for certain bacterial taxa in modulating metabolic pathways relevant to AR, warranting further validation in larger cohorts.

## Discussion

The present study elucidates the intricate interplay between gut microbial dysbiosis and metabolic perturbations in AR pathogenesis. Our multi-omics analysis revealed an AR-associated gut microbiome profile characterized by depletion of SCFA-producing genera (e.g., *Faecalibacterium*, *Ruminococcus*) and expansion of pro-inflammatory taxa (e.g., *Fusobacterium*, *Collinsella*). This dysbiosis was paralleled by compromised microbial metabolic pathways, notably SCFA biosynthesis, tryptophan metabolism (e.g., indolepyruvate), and vitamin B5 (pantothenate) and CoA biosynthesis. These findings align with the emerging paradigm of the “gut-nose axis,” wherein gut-derived microbial metabolites modulate nasal mucosal immunity through mechanisms involving AhR activation, SCFA-mediated Treg differentiation, and lipid mediator-driven inflammation (Chen et al., 2022; Lu et al., 2025; Tang et al., 2025; Wang et al., 2024).

Although gut dysbiosis has been reported in various respiratory and systemic diseases—such as asthma, COPD, and inflammatory bowel disease (Ahmadi et al., 2025; Bhutta et al., 2024; Goyal et al., 2025; Zhang H. et al., 2025; Zhu et al., 2025)—the specific microbial and metabolic signatures we identified in AR may reflect a disease-specific ecological and functional shift. For instance, the co-depletion of *Faecalibacterium* and *Ruminococcus* alongside elevated *Fusobacterium* and *Collinsella*, combined with disruptions in SCFA and phenolic acid metabolism, suggests a unique gut-nose axis profile in AR. This pattern differs from the dysbiosis seen in asthma, which often involves distinct taxa such as *Haemophilus* or *Moraxella*, and different metabolic pathways such as bile acid or sphingolipid metabolism (Hufnagl et al., 2020). Thus, while some features may overlap, the concerted changes in both taxonomy and metabolism support the potential for microbiota-targeted therapies specifically tailored for AR.

At the phylum level, we observed a notable increase in the relative abundance of *Fusobacteriota*, a phylum often associated with pro-inflammatory states, alongside a significant reduction in *Verrucomicrobiota*, which includes important short-chain fatty acid (SCFA)-producing species like *Akkermansia muciniphila*. This finding suggests a compromised capacity for microbial-mediated immunoregulation in AR patients. At the genus level, the depletion of key SCFA producers such as *Faecalibacterium, Ruminococcus*, and *Roseburia* in the AR group further underscores this notion. Conversely, the enrichment of genera like *Fusobacterium* and *Collinsella* in AR patients, taxa implicated in promoting inflammation and gut barrier dysfunction, points to a state of gut microbial dysbiosis characterized by a reduction in beneficial, immunoregulatory bacteria and an expansion of potentially pathobiontic taxa. These specific alterations provide a microbial basis for the systemic immune dysregulation observed in AR.

Interestingly, while the structure of the microbial community differed significantly between groups, its overall richness and diversity (alpha diversity) remained comparable. This indicates that AR-associated dysbiosis is not a matter of simple biodiversity loss but rather a specific rearrangement of microbial populations. The Chao1, Shannon, and Simpson indices showed no significant differences, indicating that species richness and evenness were largely unchanged, which is consistent with the findings of many previous studies (Li J. et al., 2025; Li M. et al., 2025; Ma et al., 2025). However, the near-significant reduction in phylogenetic diversity (PD whole tree, *P* = 0.051) hints at a potential loss of evolutionary complexity that warrants investigation in larger cohorts. In contrast, beta diversity analysis unequivocally demonstrated significant separation between AR and HC groups based on Bray-Curtis, Jaccard, and unweighted UniFrac distances. The significance of unweighted (qualitative) but not weighted (quantitative) UniFrac distances indicates that the compositional differences are driven primarily by the presence or absence of low-abundance (rare) taxa, rather than by changes in the relative abundance of the most common species (Tang et al., 2025; Zhang Y. et al., 2025). This subtlety highlights the importance of analyzing community structure beyond mere diversity indices to uncover clinically relevant dysbiosis.

The gut metabolome, as a functional readout of microbial activity, exhibited pronounced disturbances in AR patients. Our untargeted metabolomics approach identified several metabolites with high discriminatory power, such as Maltol and 4-Coumaric acid. Pathway enrichment analysis revealed significant alterations in critical metabolic pathways, including unsaturated fatty acid biosynthesis, steroid hormone metabolism, and central energy metabolism pathways like glycolysis and pyruvate metabolism. These findings suggest profound shifts in host-microbiota co-metabolism that could influence immune cell function and inflammatory responses. Crucially, our integrated analysis revealed significant correlations between specific bacterial genera and metabolites. The positive correlation of the dipeptide Asp-Glu and phenolic compounds like 2-methoxy-4-vinylphenol with SCFA-producing genera (*Ruminococcus*, *Faecalibacterium*) suggests a link between these beneficial bacteria and the production of immunomodulatory or barrier-strengthening metabolites. Conversely, the negative correlation between 2-methoxy-4-vinylphenol and *Ruminococcus torques*, a species sometimes associated with gut inflammation, reinforces the concept of functional antagonism within the microbial community. These microbiome-metabolite interactions provide mechanistic insights into how gut dysbiosis may contribute to AR pathophysiology through the production or modulation of specific bioactive molecules.

Our study describes associations between gut microbial dysbiosis, metabolic disturbances, and AR. However, the pathogenic impact of these microbial changes is likely indirect and contingent upon the host’s overall health and nutritional status (Essilfie et al., 2025; Zhang et al., 2024). The depletion of SCFA-producers we observed, for instance, would be particularly detrimental in a host with a diet low in fermentable fiber—the essential substrate for bacterial SCFA generation (Shin et al., 2023; van der Hee and Wells, 2021). Conversely, a fiber-rich diet might bolster resilience against such microbial loss. Similarly, the disruption in vitamin B5 (pantothenate) metabolism could be compounded by inadequate dietary intake of this vitamin, which is crucial for energy metabolism and immune function. Furthermore, the host’s pre-existing immune and metabolic state, potentially influenced by factors like vitamin D status, stress, or early-life microbial exposures, sets the threshold for how the immune system interprets metabolites like indolepyruvate (AhR ligand) or the lack of anti-inflammatory SCFAs. Therefore, the AR-associated gut profile we identified might be best viewed as a risk factor whose clinical manifestation is ultimately determined by a complex dialogue between these gut-derived signals and the host’s physiological context. Future studies integrating dietary records, nutritional biomarkers, and host genotyping with multi-omics data will be crucial to unravel these individual-specific interactions.

While our study provides evidence linking gut microbiome dysbiosis and metabolic dysfunction to AR, we acknowledge the crucial and potentially initiating role of microbiomes at the sites of allergen exposure—namely the oral and nasopharyngeal cavities. A growing body of literature indicates that dysbiosis of the upper airway microbiome is associated with AR susceptibility and severity (Pérez-Losada et al., 2023, 2024; Teng et al., 2024; Wang et al., 2023b). For instance, alterations in the nasal microbiota composition may disrupt local immune homeostasis and barrier integrity, facilitating a Th2-polarized inflammatory response to allergens (Chun et al., 2021; Salzano et al., 2018; Zeng and Liang, 2022). This raises the compelling question of how the gut microbiome, a remote site, interacts with these local microbial communities. The concept of the “gut-lung axis” or “gut-nose axis” provides a framework for understanding this cross-talk (Chioma et al., 2021; Druszczynska et al., 2024; Özçam and Lynch, 2024; Sun et al., 2024). We speculate that the upper airway microbiota may serve as the primary trigger for AR, while the gut microbiome, through the systemic release of microbial metabolites, acts as a critical immunomodulatory modulator. The gut-derived metabolites we identified—such as SCFAs, which are known to promote regulatory T-cell function and strengthen epithelial barriers, and various phenolic acids—could either suppress or exacerbate the inflammatory signals originating from the nasopharynx (Chiu et al., 2024; Hou et al., 2024; Xu et al., 2025). Therefore, the gut microbial dysbiosis we observed in AR patients might not be the initial cause but could create a pro-inflammatory systemic environment that amplifies the adverse responses initiated by the upper airway microbiome. Future studies that concurrently analyze the microbiome and metabolome from multiple sites (gut, oral, nasal) in the same individuals are essential to unravel the temporal and spatial dynamics of this interplay and to determine the primary site of dysfunction in AR.

Our study focused on bacterial communities and their metabolic outputs. However, we acknowledge that the gut microbiome comprises other kingdoms, including viruses (particularly bacteriophages) and fungi, which were not captured by our 16S rRNA sequencing approach. Bacteriophages can profoundly influence bacterial community structure and function through predation and lysogeny (Gogokhia et al., 2019; Hsu et al., 2019; Mills et al., 2013), potentially contributing to the dysbiosis we observed, such as the reduction of beneficial SCFA-producing taxa. Similarly, the gut mycobiome can modulate host immunity and interact with bacterial communities, potentially influencing inflammatory processes relevant to AR (Yang J. et al., 2023; Zhao et al., 2023). The metabolic perturbations identified in our study represent the integrated output of the entire gut ecosystem, including potential contributions from these non-bacterial components. Future studies employing shotgun metagenomics to characterize the virome and mycobiome, alongside metabolomics, are warranted to fully understand the multi-kingdom interactions within the gut-nose axis in AR pathogenesis.

Despite the advantages of our multi-omics approach, this study has several limitations. First, the relatively modest sample size may have limited the power to detect subtle microbial-metabolite associations and rendered some trends, such as the near-significant reduction in phylogenetic diversity, inconclusive. Second, our analysis focused primarily on bacterial communities and their metabolic outputs, thereby overlooking the potential contributions of other kingdoms, such as viruses (e.g., bacteriophages) and fungi, within the gut-nose axis. Furthermore, the absence of concurrent profiling of the upper airway (oral and nasopharyngeal) microbiome restricts a holistic understanding of the cross-talk between multi-kingdom microbiota across different body sites in AR. Third, the cross-sectional nature of our design precludes any causal inference regarding the observed shifts in gut microbial composition and metabolism relative to AR pathogenesis. Furthermore, it‘s important to note that our study cohort consisted exclusively of adults; future comparative studies across different age groups, particularly pediatric populations, will be essential to determine if the microbial and metabolic signatures identified here are age-specific or universal hallmarks of AR. Finally, the lack of key immunological data, including fecal sIgA levels and serum cytokine profiles, impedes a deeper mechanistic elucidation of how the identified microbial and metabolic signatures influence host systemic and mucosal immunity.

To address these limitations and advance the field, we propose several targeted directions for future research. (1) Multi-center, large-scale longitudinal studies incorporating detailed dietary records are essential to validate our findings, control for confounders, and establish temporal relationships. (2) Employing shotgun metagenomics to conduct integrated multi-kingdom (bacterial, viral, fungal) analyses of both the gut and upper airway microbiomes in the same individuals will provide a more comprehensive view of microbial ecology in AR. (3) Mechanistic causality and underlying pathways should be investigated using gnotobiotic mouse models colonized with microbiota from AR patients. (4) Interventional clinical trials, such as fecal microbiota transplantation or pre/probiotic supplementation, are warranted to assess the therapeutic potential of modulating the gut microbiome for improving clinical and metabolic outcomes in AR. The application of emerging technologies, including spatially resolved metabolomics and single-cell microbial sequencing, will further help delineate the precise spatial and functional host-microbe interactions in AR pathogenesis.

## Conclusion

In summary, this integrated multi-omics study reveals a distinct gut microbial and metabolic signature in patients with allergic rhinitis, characterized by reduced phylogenetic diversity, depletion of SCFA-producing bacteria, and dysregulation of key immunomodulatory metabolites. The correlation between specific microbial taxa and metabolic pathways underscores the potential role of gut-derived metabolites in influencing nasal mucosal immunity through systemic mechanisms. Our findings support the concept of a “gut-nose axis” in AR pathogenesis and highlight the potential for microbiota-directed interventions, such as probiotics or SCFA supplementation, to restore immune homeostasis. Future studies incorporating longitudinal design, multi-site microbiome sampling, and functional validation are needed to establish causality and translate these insights into clinical applications.

## Data Availability

The datasets supporting this study are available in public repositories. 16S rRNA sequencing data are available in the NCBI SRA under accession number PRJNA1314442. Metabolomics data are available in the MetaboLights database under accession number MTBLS13021.
