# Supplementary material for: Integrated gut microbiome and metabolomics analysis reveals microbial-metabolic cross-talk in allergic rhinitis
Source: Front Microbiol. 2025 Nov 12;16:1652915. doi: 10.3389/fmicb.2025.1652915 (PMC12646992; doi:10.3389/fmicb.2025.1652915)
Supplement: Supplementary file 1 [file Supplementary_file_1.docx]

Supplementary Material

Integrated Gut Microbiome and Metabolomics Analysis Reveals Microbial-Metabolic Cross-talk in Allergic Rhinitis

Shouyan Zhao^2,#^, Chong Xu^1#^, Min Zhang^1^, Denghan Hou^1^, Guangchen Sun^1,3*^, Ruonan Chai^1,2,3*^

^1^ Department of Respiratory Medicine, General Hospital of Northern Theater Command, Shenyang 110000, Liaoning Province, China

^2^ No. 962 Hospital of the PLA Joint Logistics Support Force, Harbin, 150000, Heilongjiang Province, China

^3^ College of Medicine and Biological Information Engineering, Northeastern University, Shenyang 110000, Liaoning Province, China

^#^These authors have contributed equally to this work

*Correspondence: Ruonan Chai, Email: lilypad_ff@126.com; Guangchen Sun, Email: [sgconcise@126.com](mailto:sgconcise@126.com)


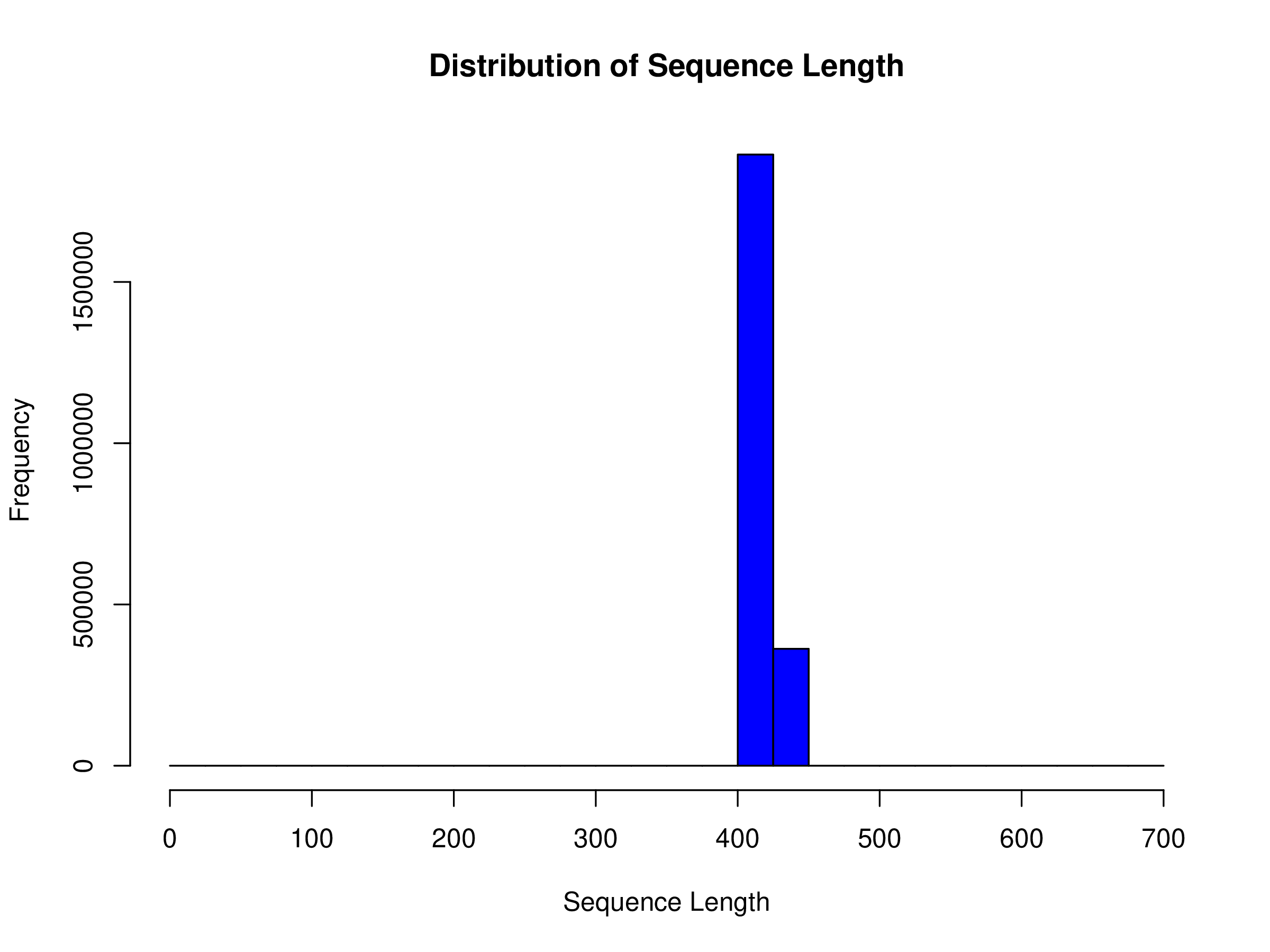


**Supplementary Figure S1.** Effective sequence length distribution.


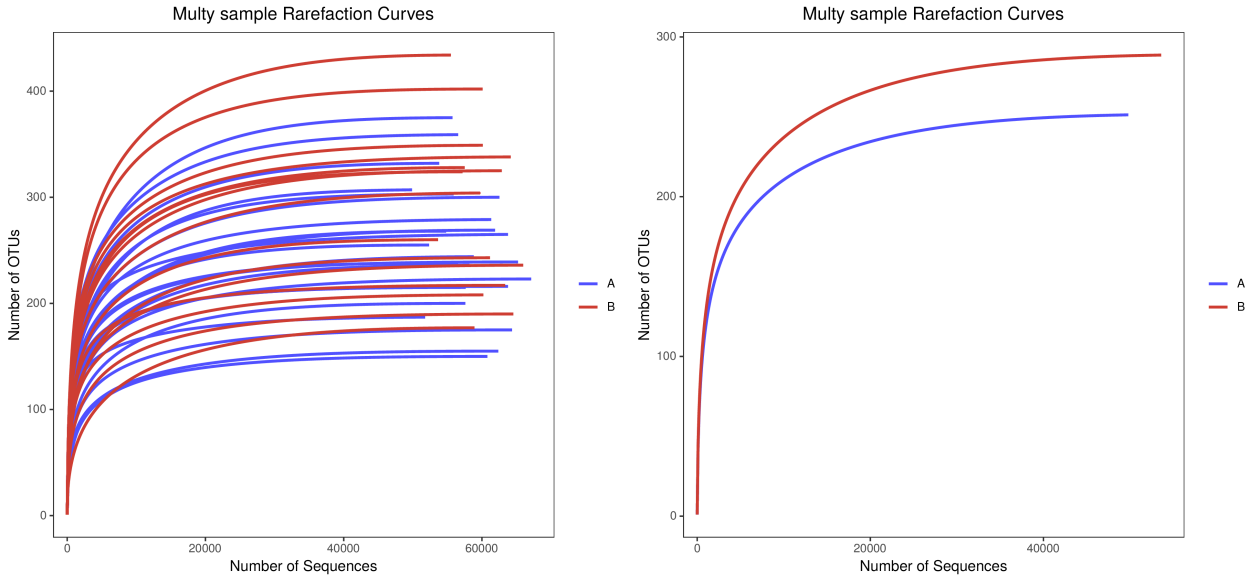


**Supplementary Figure S2.** Rarefaction curves stratified by group.


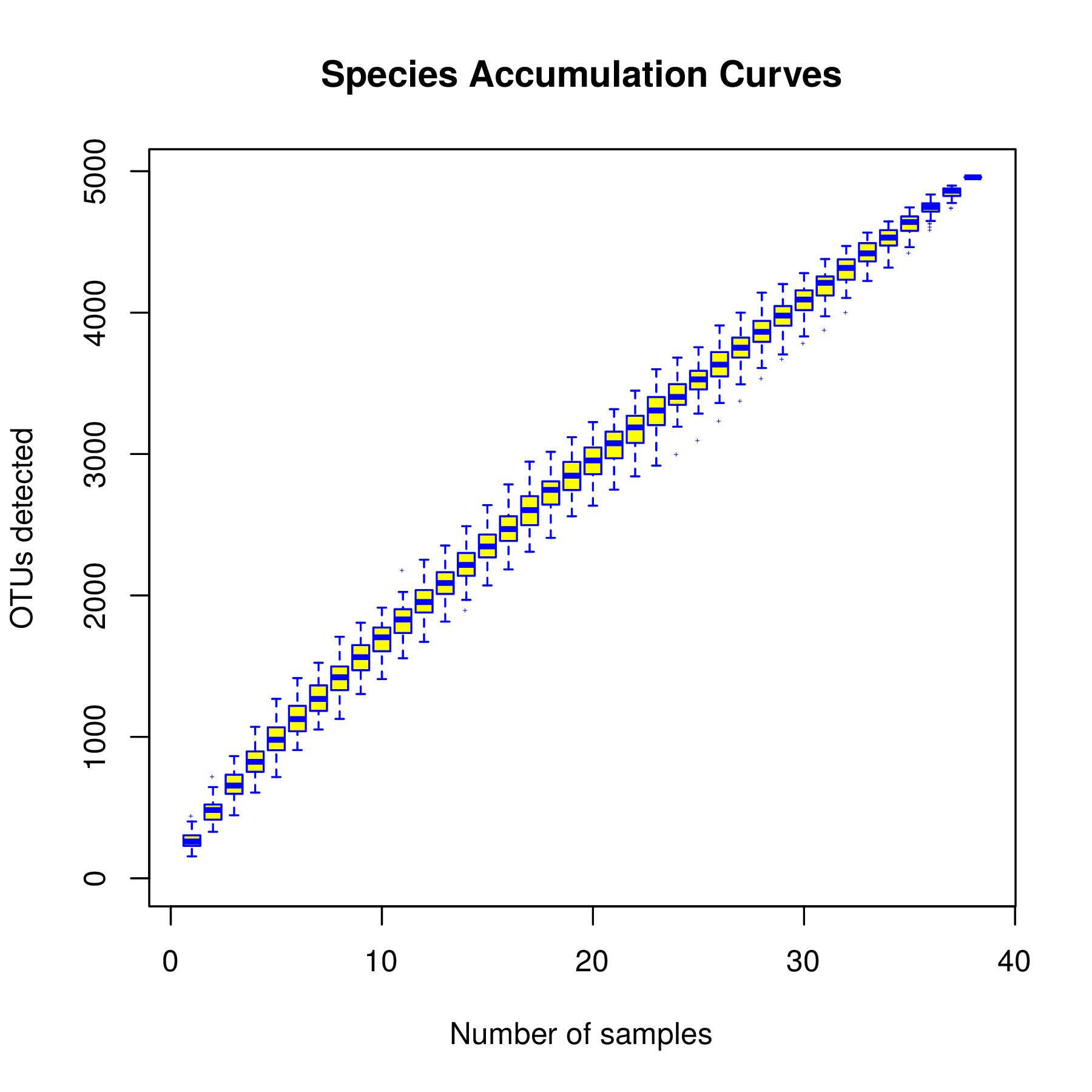


**Supplementary Figure S3.** Species accumulation curves.


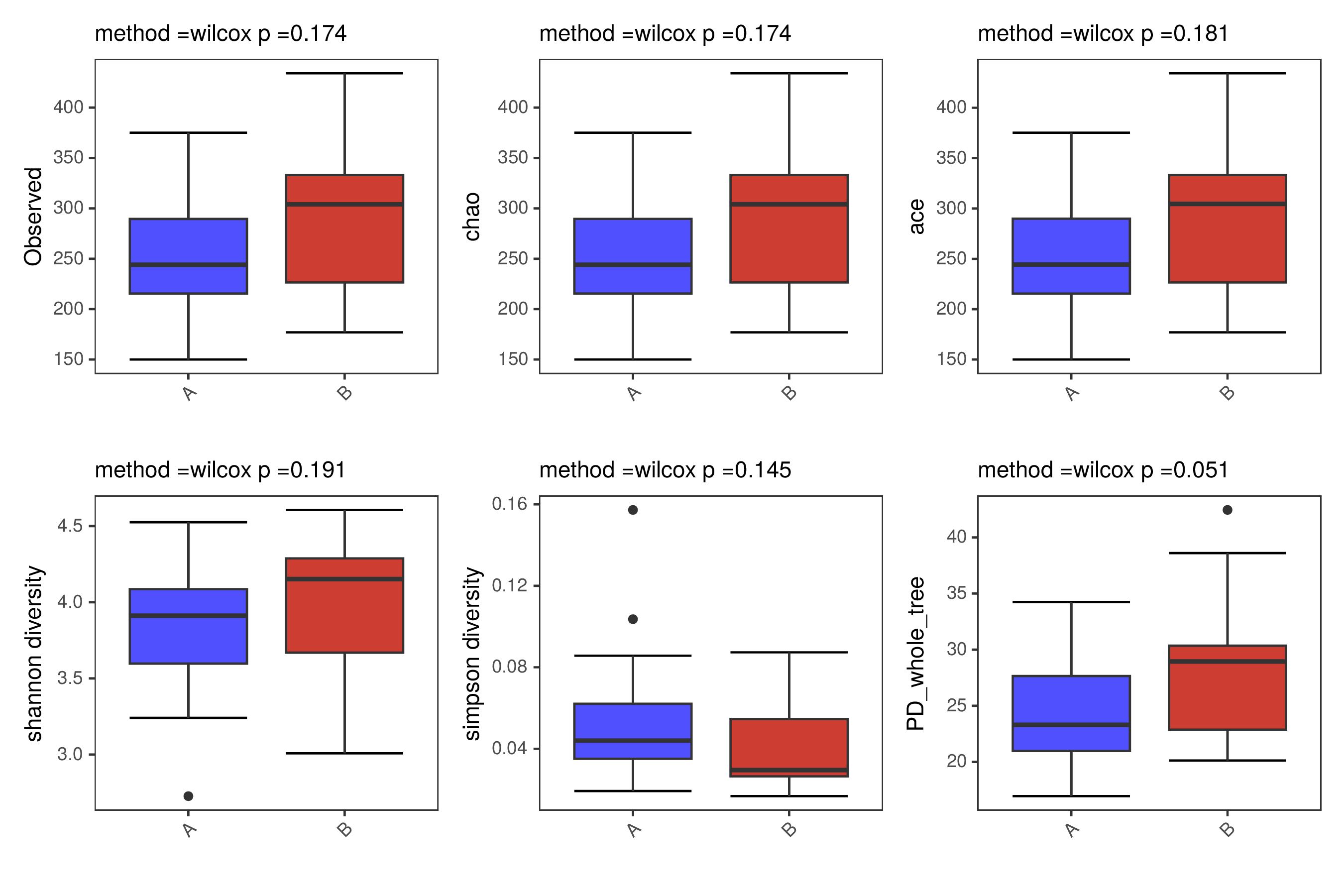
**Supplementary Figure S4.** **Alpha Diversity Box Plot Overview.**

Boxplots comparing alpha diversity indices between Allergic Rhinitis (AR) patients and Healthy Controls (HC). Indices include Observed species, Chao1, ACE (reflecting species richness), Shannon, Simpson (reflecting species diversity and evenness), and Phylogenetic Diversity (PD) whole tree. The P-values were obtained using the Mann-Whitney U test. No significant differences were observed between groups (all P > 0.05).

| **Analysis Item** | **Software and Version** | **Link** |
| --- | --- | --- |
| Quality Assessment (QA) | R 3.6.3 |  |
| Quality Control (QC) PCA Plot | ropls version 1.18.0 | [https://bioconductor.org/packages/release/bioc/html/ropls.html](https://bioconductor.org/packages/release/bioc/html/ropls.html) |
| Hierarchical Clustering Heatmap Analysis of Metabolites | pheatmap version 1.0.12 | [https://cran.r-project.org/web/packages/pheatmap/](https://cran.r-project.org/web/packages/pheatmap/) |
| Sample Dendrogram Analysis | stats version 3.6.3 (hclust function) dendextend version 1.12.0 (visualization) | [https://cran.r-project.org/web/packages/dendextend/](https://cran.r-project.org/web/packages/dendextend/) |
| Multivariate Statistical Analysis - PCA | ropls version 1.18.0 | [https://bioconductor.org/packages/release/bioc/html/ropls.html](https://bioconductor.org/packages/release/bioc/html/ropls.html) |
| Multivariate Statistical Analysis - PLS-DA | ropls version 1.18.0 | [https://bioconductor.org/packages/release/bioc/html/ropls.html](https://bioconductor.org/packages/release/bioc/html/ropls.html) |
| Multivariate Statistical Analysis - OPLS-DA | ropls version 1.18.0 | [https://bioconductor.org/packages/release/bioc/html/ropls.html](https://bioconductor.org/packages/release/bioc/html/ropls.html) |
| Parametric Test (Two-group) | t.test (stats) | [https://www.rdocumentation.org/packages/stats/versions/3.6.2/topics/t.test](https://www.rdocumentation.org/packages/stats/versions/3.6.2/topics/t.test) |
| Non-parametric Test (Two-group) | wilcox.test (stats) | [https://www.rdocumentation.org/packages/stats/versions/3.6.2/topics/wilcox.test](https://www.rdocumentation.org/packages/stats/versions/3.6.2/topics/wilcox.test) |
| Parametric Test (Multiple-group) | oneway.test (stats) | [https://www.rdocumentation.org/packages/stats/versions/3.6.2/topics/oneway.test](https://www.rdocumentation.org/packages/stats/versions/3.6.2/topics/oneway.test) |
| Non-parametric Test (Multiple-group) | kruskal.test (stats) | [https://www.rdocumentation.org/packages/stats/versions/3.6.2/topics/kruskal.test](https://www.rdocumentation.org/packages/stats/versions/3.6.2/topics/kruskal.test) |
| Boxplot / Barplot | ggplot2 version 3.3.5 | [https://cloud.r-project.org/web/packages/ggplot2/index.html](https://cloud.r-project.org/web/packages/ggplot2/index.html) |
| ROC Curve Analysis | pROC version 1.15.3 | [https://cloud.r-project.org/web/packages/pROC/index.html](https://cloud.r-project.org/web/packages/pROC/index.html) |
| Z-score Plot | ggplot2 version 3.3.5 | [https://cloud.r-project.org/web/packages/ggplot2/index.html](https://cloud.r-project.org/web/packages/ggplot2/index.html) |
| Hierarchical Clustering Heatmap Analysis of Differential Metabolites | pheatmap version 1.0.12 | [https://cran.r-project.org/web/packages/pheatmap/](https://cran.r-project.org/web/packages/pheatmap/) |
| Correlation Plot of Differential Metabolites | corrplot version 0.84 | [https://cloud.r-project.org/web/packages/corrplot/index.html](https://cloud.r-project.org/web/packages/corrplot/index.html) |
| Pathway Analysis of Differential Metabolites | MetaboAnalystR version 2.0.2 | [https://github.com/xia-lab/MetaboAnalystR](https://github.com/xia-lab/MetaboAnalystR) |

**Supplementary Table S1.** Software and version information for bioinformatic and statistical analyses
